# More Time and Effort, Same Curiosity: Expected Effort Does Not Impact Curiosity

**DOI:** 10.1162/OPMI.a.342

**Published:** 2026-03-15

**Authors:** Emily G. Liquin

**Affiliations:** Department of Psychology, University of New Hampshire, Durham, NH, USA

**Keywords:** curiosity, effort, information search, learning

## Abstract

Why do people feel curious about some questions but not others? Recent accounts of curiosity argue that curiosity should be highest when learning is likely to occur and likely to be rapid. However, it is less clear whether practical barriers to learning—for example, the effort required to gain information—matter for how curious we feel. In three preregistered experiments with a total of 419 participants, we test whether the expected degree of effort to obtain information impacts curiosity. In Experiments 1 and 2, we prompted adult participants to rate their curiosity about the answers to trivia questions. For each question, they were informed that they would receive the answer with minimal time and effort or with substantial time and effort. In Experiment 3, we additionally varied the probability that exerting effort would lead to information. Across studies, we found that effort affected decisions to seek information, but not self-reported curiosity. This suggests that subjective feelings of curiosity are unhindered by the practical costs of information search, though our decisions to actually pursue information are highly sensitive to these costs.

## INTRODUCTION

The last 50 years have seen dramatic changes in how people seek information. Before the widespread use of the internet, people sought answers to their questions by reading books and asking librarians—meaning that considerable time and effort was required to satisfy one’s curiosities. With the advent of cell phones and the internet, people could suddenly seek answers to their questions immediately, from nearly anywhere. Within the last several years, the informational landscape is changing again: now, generative AI tools can answer questions with a simple, easy to understand summary—taking nearly all effort out of information search. Thus, the time and effort required to gain information has decreased dramatically over time. Even now, however, learning problems vary in the time and effort needed to solve them. Some classes require many homework assignments and long readings, while others require fewer. Some questions can be answered by AI tools, while others are best answered by consulting experts (e.g., “What is the cause of my back pain?”)—a much more effortful process.

In the present research, we ask how variation in the time and effort required to gain information affects curiosity. Do people feel more curious about questions that they expect to require more or less effort (and therefore, more or less time) to answer? We begin, in the section that follows, by discussing prior research on curiosity, highlighting open questions about how expected effort might shape curiosity. Then, we report three experiments investigating how people’s self-reported curiosity is affected by the effort needed to gain information. The real-world relevance of this work is clear. If curiosity is dampened or heightened in response to effort, this may lead to new interventions to promote curiosity when it would be desirable: for example, in educational settings. Beyond this, understanding the effect of effort on curiosity helps provide new nuance to recent theories of curiosity.

### Curiosity: A Drive to Learn

Classic theories of curiosity propose that curiosity is a motivational state that motivates information search. For example, the “information gap” account of curiosity (Loewenstein, 1994) argues that curiosity arises when a person notices a salient gap between what they currently know and what they want to know. Recent accounts of curiosity have built on this foundation, arguing that curiosity is tightly linked to the process of learning. For example, Liquin and Lombrozo (2020; see also Lombrozo & Liquin, 2023) found that curiosity may be partly triggered not only by the metacognitive judgment that one’s current knowledge is lacking, but also by the judgment that learning is likely to occur through additional exploration. Relatedly, Metcalfe and colleagues have argued that a person is likely to experience curiosity when they feel they are on the verge of learning (Metcalfe et al., 2020, 2023). Thus, curiosity may be triggered by expectations about future learning.

Another example comes from the “learning progress” account, which proposes that the rate at which one’s predictive model of the world improves during learning—termed “learning progress”—is a key trigger of curiosity (Gottlieb et al., 2013; Poli et al., 2024). The learning progress hypothesis has roots in artificial intelligence and robotics, where it has been used to design artificial agents that bootstrap new knowledge based on self-guided exploration (Kaplan & Oudeyer, 2004; Oudeyer et al., 2007). More recently, research has shown that learning progress also affects human exploration (Poli et al., 2020, 2022, 2025; Sayalı et al., 2023; Ten et al., 2021).

Thus, to summarize, there is growing evidence that curiosity is in some way related to the *process* of learning—not just a static estimate of one’s current knowledge. There is disagreement about the exact quantity curiosity tracks: whether expected learning, learning progress, an information gap, or something else. Moreover, there is debate as to whether the triggers of curiosity can all be subsumed under a single account (e.g., the learning progress account; see Ten et al., 2025) or whether multiple triggers have a unique influence on curiosity (Liquin & Lombrozo, 2020; Poli et al., 2024). However, curiosity is clearly closely tied to the process of learning.

### Curiosity and Effort

If curiosity is somehow tied to the process of learning, we might expect that practical barriers to learning—for example, whether a high degree of effort is required to obtain novel information—might likewise matter for curiosity. For example, curiosity might differ depending on whether obtaining the relevant information requires a quick internet search or driving to the library to search in a physical book. Does this variation in the effort required to gain information, when disentangled from learning progress and expected learning, affect curiosity? Based on prior work, we may expect two contradictory possibilities: that high levels of expected effort might dampen curiosity, or that high levels of expected effort might not affect—or even enhance—curiosity.

First, expected effort might dampen curiosity. If the prospect of exerting effort is generally demotivating, we might expect curiosity—often thought of as a motivational or drive-like state (see Kidd & Hayden, 2015; Loewenstein, 1994; Silvia, 2012)—to decrease when effort is required. Moreover, recent accounts of curiosity (FitzGibbon et al., 2020; Gruber et al., 2014; Lau et al., 2020; Marvin & Shohamy, 2016) have proposed that information is akin to reward, and curiosity is a motivational pull towards the reward of gaining information. For other rewards, prior work has documented “delay discounting” and “effort discounting”: people value rewards less when more time or effort is required to obtain them (Ainslie, 1975; Frederick et al., 2002; Hull, 1943; Prévost et al., 2010; Rachlin et al., 1991; Westbrook et al., 2013). If curiosity is a form of reward anticipation, we might expect delay discounting and effort discounting for curiosity, as well. Specifically, people should be less curious when high levels of effort are needed to gain information relative to lower levels of effort. Consistent with this, prior work has shown that people are less likely to seek information when it will be delivered after a delay rather than immediately (Molnar & Golman, 2024). However, other work has shown that subjective self-reports of curiosity are *not* impacted by the length of delay before obtaining new information (Noordewier & van Dijk, 2017). Thus, it is unclear whether delay discounting and effort discounting extend to curiosity. If they do, we would predict lower levels of curiosity when more effort is expected.

However, there are also reasons to expect that expected effort might not affect curiosity, or perhaps even enhance it. Children persist *less* in seeking information when information will be provided regardless of their efforts (Rett & Walker, 2020)—perhaps suggesting lower levels of curiosity when little effort is needed to obtain information. Moreover, there is evidence that curiosity can motivate learners to go to great lengths to gain information. For example, higher curiosity is associated with more willingness to spend time (Dubey & Griffiths, 2020; Kang et al., 2009) and exert cognitive effort (Spitzer et al., 2025) to gain the answer to a trivia question. People will even subject themselves to electric shocks to receive information when curious (Hsee & Ruan, 2016; Lau et al., 2020). This suggests that curiosity might *not* be dampened when effort is required to learn—instead, curiosity might motivate learners even in these circumstances.

In summary, it is not clear whether we should expect an effect of effort on curiosity. If curiosity is a form of reward anticipation, we would expect lower levels of curiosity when more effort is needed to gain information—consistent with delay discounting and effort discounting. However, curiosity motivates people to approach effortful and time-consuming tasks to obtain new information, perhaps suggesting that curiosity is maintained or even enhanced in the face of high expected effort. Beyond shedding light on curiosity’s underlying mechanisms, studying how expected effort affects curiosity has practical implications: if we can understand what elicits curiosity, we may be able to induce learners’ curiosity when it would be desirable (e.g., in the classroom). Curiosity has many benefits—for example, curiosity motivates information search and enhances memory (Gruber et al., 2014; Kang et al., 2009). As a result, it is critical to understand what motivates (or dampens) curiosity itself, especially in a world where technological innovations provide increasingly effortless access to information.

### The Present Research

In the present research, we investigate whether expected effort affects curiosity in three experiments. To test this, we measure participants’ curiosity about trivia questions (e.g., “Which is the hottest planet in the solar system?”) after telling them that they will need to exert high levels of effort or low levels of effort to reveal the answer. We manipulate the effort associated with information using physical effort (15 vs. 60 key presses to reveal an answer) in Experiments 1–2 and cognitive effort (solve a word search puzzle to reveal an answer) in Experiment 3. In Experiments 1–2, the participant always expects the answer to be received—but the amount of effort (and time) required to receive the answer varies. In Experiment 3, we include the possibility that the participant’s efforts will fail: the participant only sees the answer to the trivia question if they are able to solve an effortful task within a fixed time limit.

Though the trivia-question paradigm does not resemble many aspects of real-world curiosity—for example, questions are supplied to participants rather than spontaneously generated—this is a common paradigm in studies of curiosity (e.g., Gruber et al., 2014; Kang et al., 2009; Metcalfe et al., 2021; Wade & Kidd, 2019). Moreover, the simplicity and brevity of trivia questions allow us to capture a substantial amount of data from each participant, increasing statistical power. That said, in the General Discussion, we include further discussion of how our results might generalize to more realistic learning tasks.

In all studies, we measure subjective feelings of curiosity through self-report. We also measure participants’ confidence that they already know the answer to each question. In prior work, curiosity is strongly related to confidence, with the highest levels of curiosity at moderate levels of confidence (Dubey & Griffiths, 2020; Hsiung et al., 2023; Kang et al., 2009; Metcalfe et al., 2023; Spitzer et al., 2024; Ten et al., 2025). Moreover, confidence in one’s own knowledge can be inflated when information is easily accessible with little effort (e.g., through internet search; see Fisher et al., 2015). Thus, if effort impacts curiosity, this could be because effort leads to inflated confidence. Moreover, effort might only impact curiosity about a question when participants are *not* highly confident they already know the answer (i.e., when there is genuinely new information to be learned).

To preview our results, across all three preregistered studies, we find no evidence that expected effort influences subjective self-reports of curiosity. In fact, using Bayesian analyses, our data are much more likely under the null hypothesis—that expected effort does not affect feelings of curiosity—than the alternative hypothesis—that expected effort affects feelings of curiosity. This is not because participants were insensitive to the manipulation of expected effort. In Experiments 2–3, we add a measure of information search: we ask whether participants choose to engage in the effortful task to reveal the answer to each trivia question. Previewing our results, we find that expected effort *does* affect people’s decisions to seek information. This suggests that people are generally sensitive to the manipulation of effort, even though their subjective feelings of curiosity fail to respond. In sum, people continue to feel curious even when they are unlikely to receive the desired information easily—or at all.

## EXPERIMENT 1

In Experiment 1, we conducted an initial test of whether expected effort impacts curiosity. We manipulated how much effort would be required to gain new information (the answer to a trivia question), and we tested how this affected participants’ reported curiosity. Experiment 1 was preregistered at https://aspredicted.org/z525-3njf.pdf. All data and analysis code (for all experiments) are available at https://osf.io/js7x2.

### Methods

#### Participants.

We recruited 82 participants from a student participant pool at the University of New Hampshire. All participants completed the study online. An additional 18 participants were excluded for failing to pass three attention checks (described below). The sample size for this study was determined based on available resources for data collection. However, we conducted a post-hoc sensitivity analysis to determine the smallest effect size that we could reliably detect with 80% power with our actual sample size, using the simr R package (Green & MacLeod, 2016). This analysis revealed that we could detect a minimum true effect of *β* = 0.09 with 80% power. Therefore, we were well-powered to detect a small effect.

Our participants were college-aged, ranging from 18–24 years of age (*M* = 19). 74% of participants identified as women, 24% as men, and 1% as non-binary or genderqueer (1 participant unspecified). Most participants (94%) were White and Non-Hispanic/Latinx.

In all experiments, participants provided informed consent prior to participating. All experiments were approved by the Institutional Review Board for the Protection of Human Subjects in Research at the University of New Hampshire.

#### Materials.

The materials were 40 general knowledge trivia questions from Metcalfe et al. (2021). These questions were originally from the Nelson and Narens’ norms (Nelson & Narens, 1980) and were modified by Bloom et al. (2018). All trivia questions had one-word answers and were presented in a random order for each participant.

#### Procedure.

**Figure 1.**
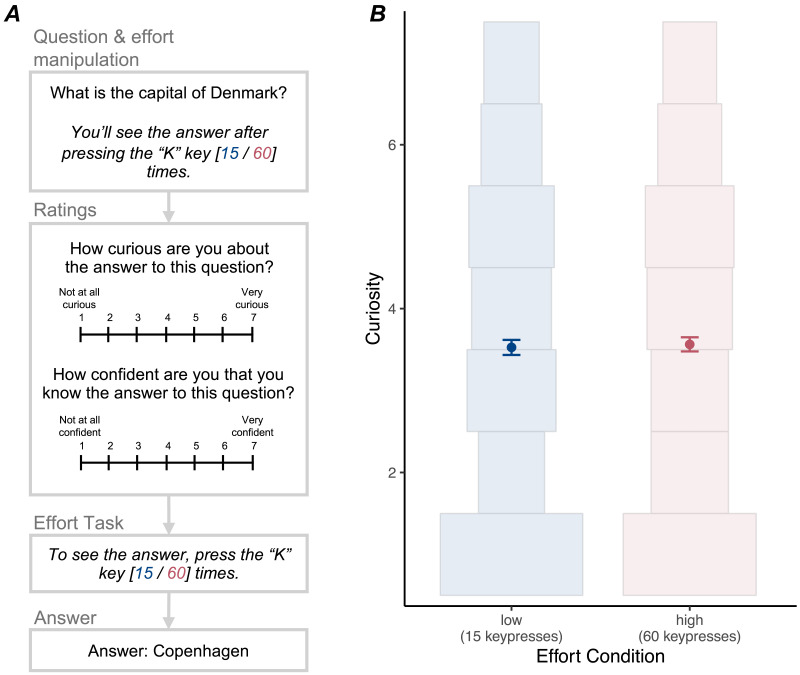
Experimental paradigm (A) and results (B) for Experiment 1. (B) Points indicate mean curiosity in each effort condition; error bars indicate 95% CIs. Histograms show the distribution of curiosity ratings within each effort condition. There was no evidence that effort condition affected curiosity.

All participants completed 40 trivia trials. On each trial (see Figure 1), participants saw a single trivia question, with an associated effort condition (manipulated within-subjects). In the low-effort condition (20 questions), participants were informed that they would need to press the “K” key on their keyboard 15 times to see the answer. In the high-effort condition (20 questions), participants were informed that they would need to press the “K” key 60 times to see the answer. We randomly assigned an effort condition to each question independently for each participant, meaning each question was seen by some participants as a low-effort question and other participants as a high-effort question. Notably, in both cases, participants needed to exert *some* amount of effort to see the answer. This reduces possible confounds: in both conditions, participants expect to complete a task (which we call the “effort task”) to receive the answer, beyond just clicking a single key or button. Moreover, in both cases, participants can see measurable progress towards completing the task (a countdown that decrements as the key is pressed, see below).

After participants viewed the question and the associated expected effort condition (but before beginning the effort task), they were prompted to rate curiosity (“How curious are you about the answer to this question?” 1 = Not at all curious, 7 = Very curious) and confidence (“How confident are you that you know the answer to this question?” 1 = Not at all confident, 7 = Very confident). The order of these questions was randomized for each participant (but remained consistent across the 40 trials).

After these responses, participants completed the effort task by pressing the “K” key the required number of times, then were shown the answer. Note that participants were required to complete the effort task and reveal the answer on each trial. This was to ensure that any effect of the effort manipulation on subjective curiosity could not be explained by participants’ preemptive decision to opt out on high-effort trials. However, in Experiment 2, we include a condition where participants have the option to opt out of the effort task on each trial.

Upon receiving the answer, participants rated answer satisfaction (“How satisfied are you by the answer?” 1 = Not at all satisfied, 7 = Very satisfied). This measure was exploratory, and analyses are reported in the Supplementary Materials.

This procedure was repeated for 40 trivia questions (see Supplementary Materials for exploratory analyses of change over trials). On the final (41st) trial, participants completed an attention check: they saw the same curiosity and confidence ratings but were prompted to respond with a particular value (6). This trial was otherwise visually identical to all preceding trials, and participants had no indication that this was the final trial. Finally, participants reported demographic information and completed a final attention check (typing a specified word in a textbox).

#### Analytic Approach.

Prior to all analyses, we *z*-scored curiosity and confidence (standardizing across the entire dataset). Expected effort was contrast coded, with the high-effort condition coded as −0.5 (reference group) and the low-effort condition coded as 0.5. In all analyses reported below, we use mixed-effects models with by-participant random slopes and intercepts and by-trivia-question random intercepts. We report regression coefficients with 95% confidence intervals. In addition, we estimate the statistical significance of fixed effects using likelihood ratio tests comparing a model with a fixed effect for the term of interest to a model without that effect.

Analyses were conducted using R Statistical Software (v4.3.2; R Core Team, 2023). We used the lme4 package (Bates et al., 2015) and car package (Fox & Weisberg, 2019) for mixed-effects models and the rstanarm package (Goodrich et al., 2020) and bayestestR package (Makowski et al., 2019) for Bayesian models.

### Results

#### Manipulation Check.

As predicted, participants spent longer on the effort task in the high-effort condition compared to the low-effort condition. For this analysis only, we removed 23 outlier trials (out of 3280 total trials across 82 participants), where a participant spent longer than 23.11 seconds pressing the “K” key (third quartile plus 1.5 times the interquartile range). Then, we fit a mixed-effects model predicting time, with effort condition as a fixed effect. There was a significant effect of effort condition, *b* = −8.08, 95% CI [−8.34, −7.82], *χ*^2^(1) = 315.25, *p* < .001. The average time a participant spent on low-effort trials was 3.44 seconds (*SD* = 0.92), versus an average of 11.50 seconds (*SD* = 1.79) on high-effort trials. This analysis was not preregistered, but it validates that we successfully manipulated effort (measured via time) through our effort task.

#### Effort and Curiosity.

We found no evidence that curiosity was affected by effort condition (see Figure 1). We fit a mixed-effects regression model predicting curiosity, with effort condition as a fixed effect. There was no evidence for an effect of effort on curiosity, *β* = −0.02, 95% CI [−0.08, 0.04], *χ*^2^(1) = 0.26, *p* = .61. Participants’ mean curiosity rating was 3.53 (*SD* = 1.11) in the low-effort condition and 3.56 (*SD* = 1.06) in the high-effort condition. Thus, participants’ curiosity did not appear to vary as a function of how quickly and easily they could receive the answer.

To further support this conclusion, we conducted an exploratory Bayesian version of the above analysis.^1^ We estimated the Bayes Factor in favor of a null model that included a fixed intercept, by-participant and by-trivia-question random intercepts, and a by-participant random slope for effort condition, compared to a full model that additionally included a fixed effect of effort condition. This comparison tests for a population-level effect of effort condition, while accounting for individual variation in the effect. The Bayes Factor in favor of the null model (BF = 133.37) suggested that the null model provided much better predictions of the data than the full model. In addition, the posterior estimate for the fixed effect of effort in the full model was concentrated around zero (median = −0.02, 95% HDI [−0.08, 0.04]). Together, this suggests that a population-level effect of expected effort on curiosity is unlikely.

We preregistered that we would test whether confidence mediated the effect of expected effort on curiosity. Since we did not find an effect of expected effort on curiosity, this analysis was not conducted. However, we report additional analyses in the Supplementary Materials showing a null effect of expected effort on confidence. We also report analyses showing that the effect of effort on curiosity does not vary across levels of confidence. When participants judge high confidence that they already know the answer, they are unlikely to be curious—but this cannot explain our null findings. Across levels of confidence, there was no evidence for an effect of effort on curiosity.

### Discussion

In Experiment 1, participants rated their curiosity about trivia questions, knowing that they were about to spend only a little time and effort or a lot of time and effort to receive the answer. Using Bayesian analyses, the data were more likely under the null hypothesis than the alternative hypothesis, suggesting that there was no overall effect of expected effort on curiosity.

That said, our task has several peculiarities compared to real-world information search. For example, participants were told directly how much effort was required on each trial but were not given the option to experience the degree of effort until after rating curiosity. In contrast, in real-world learning problems, we often experience effort first-hand: we try to seek information, then we find it to be more or less effortful. Moreover, we only measured *subjective* curiosity through self-report judgments. These self-report judgments may come apart from people’s actual decisions to seek information. In particular, given that people generally avoid high-effort tasks (see e.g., Hartmann et al., 2013; Kool et al., 2010; Kurzban et al., 2013; Westbrook & Braver, 2015), we predict that participants should be less likely to seek information when information search is likely to be effortful. We address these limitations in Experiment 2.

## EXPERIMENT 2

In Experiment 2, we test whether effort affects curiosity when participants have already experienced the amount of effort needed to acquire information. In many real-world learning tasks, we might expect a particular amount of effort to be required to gain new information—for example, we might know how many reading assignments a class requires. However, our experience of curiosity might shift *only after initial engagement* with these effortful tasks. To test this possibility, Experiment 2 manipulates the number of keypresses required to reveal each letter of the answer to a trivia question, and we prompt participants to rate curiosity about the answer after they have exerted effort to reveal the first letter. As a result, they have already experienced the required amount of effort at the time curiosity is assessed. Notably, we continue to tell participants the amount of effort required at the start of each trial—so exerted effort matches their expectation perfectly. However, if actual effortful engagement is required to influence curiosity, we would expect an effect of effort here but not in Experiment 1.

Experiment 2 also investigates how effort affects information search. The variations in effort tested in the present research were relatively small. In Experiment 1, participants spent about 8 seconds longer on the effort task in the high-effort condition compared to the low-effort condition. It is possible that effort did not affect curiosity in Experiment 1 because the manipulation of effort was too subtle for participants to notice it. One way of ruling out this possibility is to show that the manipulation affects information search—which would suggest that participants do notice the variation in effort. To do so, we introduce a new task condition, where participants can choose whether to seek information on each trial. In the forced-effort condition (like Experiment 1), participants always exert effort to see each answer. In the choice condition (new), participants can choose whether to exert effort to see each answer. Like prior research (e.g., Dubey & Griffiths, 2020; Kang et al., 2009; Spitzer et al., 2025), information search was operationalized by participants’ willingness to spend time or exert effort to gain information in the choice condition. Notably, unlike prior research, information search cannot be interpreted as a valid measure of curiosity because the degree of effort varies systematically across trials. Information search decisions likely reflect both the benefits of information search (e.g., satisfying curiosity, which may itself be influenced by effort—the question being studied here) and the costs of information search (e.g., exerting effort). Because effort can affect information search through both pathways, an effect of effort on information search does not provide clear evidence for whether effort affects curiosity. We return to this point in the General Discussion.

Finally, we conduct this study using a new population of participants. Experiment 1 used a university participant pool and was therefore limited in diversity of age, gender, and race/ethnicity. To increase the generalizability of our results, we reach a broader sample of participants using Prolific.

Experiment 2 was preregistered at https://aspredicted.org/bb6v-xdh7.pdf.

### Methods

#### Participants.

We recruited 174 participants from Prolific, filtering recruitment for participants in the United States who had completed at least 50 tasks on Prolific with a minimum 95% approval rate. All participants completed the study online. An additional 21 participants were excluded for failing to pass three attention checks (as described in Experiment 1). We also excluded 5 participants who provided the same curiosity and/or confidence rating on every trial.

Participants were randomly assigned to one of two task conditions: the forced-effort condition (*n* = 88) or the choice condition (*n* = 86).

Our target sample size was 150. Based on power analysis using the simr R package (Green & MacLeod, 2016), a sample of 75 participants in the forced-effort condition would provide at least 80% power to detect a small effect (*β* = 0.15) of effort condition on curiosity.

Our sample ranged from 21–74 years of age (*M* = 41). 52% of participants identified as women, 44% as men, and 1% as non-binary or genderqueer (2% not specified). Participants’ race/ethnicity were reported as follows: 71% White, 13% African American/African/Black, 6% Asian/Asian American, 4% Hispanic/Latinx/Spanish Origin, 4% multiracial or multiethnic, 1% Middle Eastern/North African, 1% Pacific Islander/Native Hawaiian (1 not specified).

#### Materials.

The materials were 20 general knowledge trivia questions from Metcalfe et al. (2021), most of which were distinct from those used in Experiment 1. In this experiment (unlike Experiment 1), we required that all trivia answers were five letters long. Since the answers were revealed one letter at a time, using a fixed answer length guaranteed that all low-effort answers required 15 keypresses to obtain (3 per letter), and all high-effort answers required 60 keypresses to obtain (12 per letter). Most trivia questions from Experiment 1 did not have five-letter answers, so we selected a new set of trivia questions for this experiment.

#### Procedure.

The procedure was similar to Experiment 1. In this case, participants were informed that they would need to press the “K” key a certain number of times to reveal each *letter* of the answer to each question. Participants completed 20 trials. In the low-effort condition (10 questions), participants were informed that they would need to press the “K” key 3 times to see each letter. In the high-effort condition (10 questions), participants were informed that they would need to press the “K” key 12 times to see each letter.

**Figure 2.**
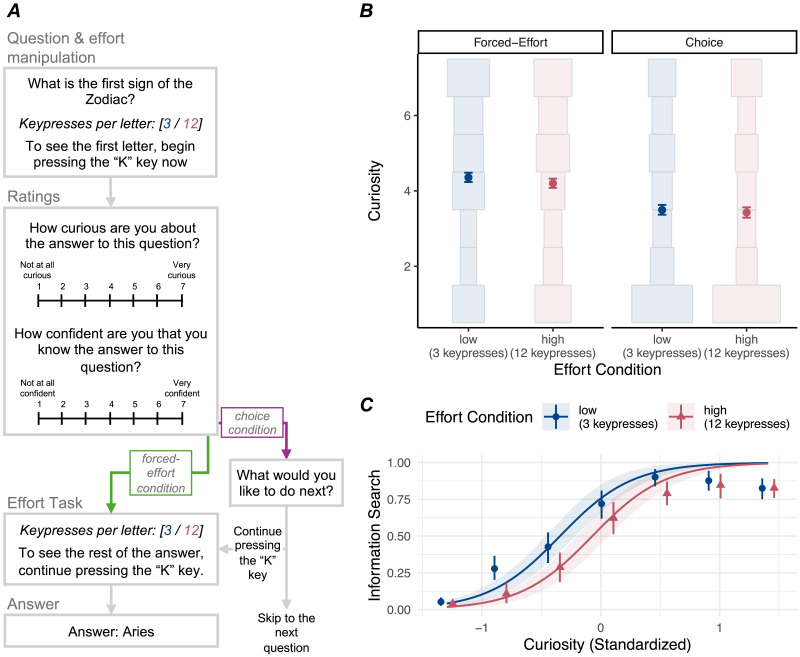
Experimental paradigm (A) and results (B, C) for Experiment 2. (B) Points indicate mean curiosity in each effort condition and task condition; error bars indicate 95% CIs. Histograms show the distribution of curiosity ratings within each effort condition and task condition. There was no evidence that effort condition affected curiosity. (C) Points indicate proportion of trials where a participant (in the choice condition) chose to engage in information search at each level of curiosity, error bars indicate 95% CIs. Lines (with 95% CIs) display model predictions from the reported mixed-effects model predicting information search as a function of curiosity and effort condition. Information search was more likely at higher levels of curiosity and lower levels of expected effort.

On each trial (see Figure 2), participants saw a single trivia question, with an associated effort condition (how many keypresses would be required for each letter). Then, they were prompted to press the “K” key the specified number of times to reveal *only the first letter* of the answer. After the first letter was revealed, participants rated curiosity and confidence (as in Experiment 1).

Then, participants in the forced-effort condition continued to press the “K” key until the entire answer was revealed. Participants in the choice condition were given the option to either continue pressing the “K” key to see the rest of the answer, or skip to the next question. In the Results, we refer to choosing to continue pressing the “K” key as “information search.”

Finally, participants completed the same attention checks and demographic questions as Experiment 1.

#### Analytic Approach.

As in Experiment 1, we *z*-scored curiosity and confidence prior to analyses (standardizing across the entire dataset). Effort was contrast coded, with the high-effort condition coded as −0.5 (reference group) and the low-effort condition coded as 0.5. Task condition was also contrast coded, with the forced-effort condition coded as −0.5 (reference group) and the choice condition coded as 0.5. In all analyses reported below, we use mixed-effects models, with by-participant random intercepts and slopes (for within-subjects variables) and by-trivia-question random intercepts. We report regression coefficients with 95% confidence intervals. In addition, we estimate the statistical significance of fixed effects using likelihood ratio tests comparing a model with a fixed effect for the term of interest to a model without that effect.

As preregistered, we first analyzed how effort condition affected curiosity in the forced-effort task condition, replicating Experiment 1. Focusing our analyses on the forced-effort task condition also ensures that any effect of the effort manipulation on subjective curiosity could not be explained by participants’ preemptive decision to opt out on high-effort trials. Then, we tested whether having the option to pursue information search (forced-effort condition vs. choice condition) moderates the effect of effort on curiosity. This allows us to test whether an effect of effort might only emerge when participants *do* have the ability to opt out of exerting effort. Finally, we tested how effort affects information search in the choice condition, validating our manipulation of effort.

### Results

#### Manipulation Check.

As in Experiment 1, we found that participants generally spent more time on the effort task in the high-effort condition than the low-effort condition. We measured how long it took participants to reveal the first letter of each answer (the first 3 or 12 keypresses), as well as how long it took participants to reveal the remainder of each answer. For the first letter, we removed 219 outlier trials (6% of trials), for this analysis only. For the remaining letters, we removed 116 outlier trials (5% of trials, excluding trials in the choice condition where participants chose not to reveal the answer). There was a significant effect of effort condition on time to reveal the first letter, *b* = −1.69, 95% CI [−1.84, −1.54], *χ*^2^(1) = 225.05, *p* < .001, and time to reveal the remaining letters, *b* = −6.54, 95% CI [−6.83, −6.25], *χ*^2^(1) = 420.86, *p* < .001. In the low-effort condition, participants revealed the first letter after an average 5.10 seconds (*SD* = 2.16) and the remaining letters after an average 4.53 seconds (*SD* = 2.39). In the high-effort condition, participants revealed the first letter after an average 6.75 seconds (*SD* = 2.16) and the remaining letters after an average 11.00 seconds (*SD* = 2.78). This analysis was exploratory, but it suggests that people took longer to reveal information in the high-effort condition than the low-effort condition—validating our manipulation.

#### Effort and Curiosity.

Next, we tested for an effect of effort on curiosity in the forced-effort condition, replicating Experiment 1. We again found no evidence that effort affected curiosity in the forced-effort condition (see Figure 2), *β* = 0.07, 95% CI [−0.005, 0.14], *χ*^2^(1) = 3.33, *p* = .07. In the low-effort condition, the average participant-level curiosity rating was 4.36 (*SD* = 1.44). In the high-effort condition, the average participant-level curiosity rating was 4.20 (*SD* = 1.43). To further test the effect of effort on curiosity, we conducted a non-preregistered Bayesian analysis. As in Experiment 1, we estimated the Bayes Factor in favor of the null model over an alternative model with a fixed effect for effort. The Bayes Factor suggested that the observed data were more likely under the null model than the alternative model (BF = 28.93). However, the posterior estimate for the fixed effect of effort in the alternative model suggested a reasonable possibility of a positive effect (median = 0.07, 95% HDI [−0.01, 0.14]). Thus, there was mixed evidence for an effect of effort on curiosity in the forced-effort condition, partly contradicting Experiment 1.

In follow-up exploratory analyses, reported in full in the Supplementary Materials, we tested whether the effect of effort on curiosity in the forced-effort condition varied across levels of confidence. Especially in this experiment, where participants rated their confidence after seeing the first letter to the answer, high confidence judgments likely imply that participants already knew the answer to the question—making their curiosity judgments difficult to interpret. Indeed, we found evidence for an interaction between curiosity and confidence, with the effect of effort on curiosity only emerging at the highest levels of confidence. As reported in the Supplementary Materials, the interaction between confidence and effort condition was also only detected in the forced-effort condition, and not in the choice condition.

As additional exploratory analyses, we repeated the above analyses while excluding any trials where a participant rated their confidence as a “7” on the 7-point scale (suggesting they already knew the answer). In this case, there was no evidence for an effect of effort on curiosity in the forced-effort condition, *β* = 0.03, 95% CI [−0.05, 0.10], *χ*^2^(1) = 0.47, *p* = .49. Moreover, in the Bayesian version of this analysis, the observed data were far more likely under the null model than the alternative model (BF = 99.92), and the posterior estimate for the fixed effect of effort was consistent with a null effect (median = 0.03, 95% HDI [−0.06, 0.11]). While these analyses were exploratory and should therefore be interpreted with caution, the results suggest that effort ordinarily has no impact on curiosity, unless the answer is already known and one has no choice but to exert effort to reveal it.

All analyses above focused only on the forced-effort condition, where participants had no choice but to engage in the effort task. Next, we investigated whether the effect of effort on curiosity varied across task conditions (forced-effort vs. choice). We fit a mixed-effects regression model predicting curiosity, with task condition and effort as fixed effects. There was no evidence for an interaction between effort and task condition, *β* = −0.03, 95% CI [−0.13, 0.08], *χ*^2^(1) = 0.31, *p* = .58. In the choice condition, the participant-level average curiosity rating was 3.49 (*SD* = 1.47) for low-effort trials and 3.43 (*SD* = 1.46) for high-effort trials (see Figure 2). Interestingly, in a model without the interaction term, there was evidence for an effect of task condition on curiosity, *β* = −0.37, 95% CI [−0.55, −0.18], *χ*^2^(1) = 14.51, *p* < .001, with lower curiosity ratings in the choice condition (*M* = 3.46, *SD* = 1.39) than the forced-effort condition (*M* = 4.28, *SD* = 1.39). Prior work has shown that choice over *which* piece of information is revealed (e.g., which lottery is played, which video is watched) can boost curiosity (Romero Verdugo et al., 2023; Schutte & Malouff, 2019). Here, in contrast, choice over *whether* a fixed piece of information would be revealed led to reduced curiosity. One possible interpretation is that self-reported curiosity is partly influenced by participants’ intention to seek information, such that participants who have already decided not to seek information (an option only available in the choice condition) report lower curiosity. Because this effect was not predicted a priori and is not replicated in Experiment 3 (see below), we interpret it cautiously. Nonetheless, it suggests that the effect of choice on curiosity warrants future study.

#### Effort and Information Search.

Finally, we tested the effect of effort on information search. On average, participants chose to seek information on 8.95 out of 20 trials (*SD* = 4.32). However, we found that participants were more likely to seek information when expecting a lower-effort task than a higher-effort task (see Figure 2). We fit a mixed-effects logistic regression model predicting information search on each trial, with effort condition and curiosity as fixed effects. There was evidence for a significant effect of effort condition, *odds ratio* (*OR*) = 2.45, 95% CI [1.57, 3.84], *χ*^2^(1) = 15.16, *p* < .001, with higher odds of information search in the low-effort condition compared to the high-effort condition. On average, participants chose to seek information on 4.87 (*SD* = 2.39) out of 10 low-effort trials and 4.08 (*SD* = 2.52) out of 10 high-effort trials. There was also an effect of curiosity, *OR* = 27.92, 95% CI [16.27, 47.92], *χ*^2^(1) = 136.91, *p* < .001, with higher curiosity predicting greater odds of information search. Of all trials where a participant provided a “1” curiosity rating (the minimum), participants chose to seek information on only 5% of trials. In contrast, of all trials where a participant provided a “7” curiosity rating (the maximum), participants chose to seek information on 83% of trials. Thus, replicating prior work, people are willing to engage in time-consuming tasks to satisfy their curiosity (e.g., Kang et al., 2009)—reflecting curiosity’s motivational force. Moreover, the level of expected effort independently predicted information search decisions.

Notably, though participants were not required to seek information on any trial, none of the participants opted out of the effort task on every trial. Four participants only sought information on one trial. Conversely, two participants sought information on every trial. This suggests that participants did not uniformly avoid effort, even though doing so would likely shorten the experiment. Most employed a mixed strategy.

### Discussion

Experiment 2 tested the effect of effort on curiosity, when participants had the opportunity to experience the degree of required effort prior to rating curiosity. Largely, we replicated the Experiment 1 finding that effort is unlikely to affect the subjective experience of curiosity. There was one exception: engaging in a low-effort task led to increased curiosity when participants were already highly confident they knew the answer, particularly when they had no choice but to continue exerting effort. We discuss this finding further in the General Discussion.

We also tested whether information search was sensitive to effort. We found strong evidence that people were more likely to seek information when information was less effortful to obtain. This demonstrates that people are sensitive to the manipulation of effort overall—and the null effect for self-reported curiosity can therefore not be explained by insensitivity to the manipulation. Importantly, because there are independent reasons to expect that effort would affect information search (e.g., the desire to avoid costly tasks, the desire to finish the experiment more quickly), finding that effort affects information search does not necessarily imply that effort affects curiosity—though we return to this point in the General Discussion.

## EXPERIMENT 3

Experiments 1–2 provide evidence against the idea that effort affects the subjective experience of curiosity. However, in both experiments, participants were guaranteed to receive information after exerting sufficient effort. In contrast, in real-world learning settings, effort may not always pay off. A person can search a library for a particular textbook but fail to find it. An internet search can be unsuccessful. A student’s visit to office hours can fail to clarify a confusing concept. In this sense, learning problems do not just vary in how much effort is needed to solve them—but also how likely they are to be solved at all. Generally, when a learning problem requires more effort to solve, it is more likely that a learner will *fail* to learn (e.g., because they fail to invest sufficient time and effort, or because the time and effort required is beyond their capacity).

In Experiment 3, we ask whether effort affects curiosity when failure is possible. Are people more curious when information search is less likely to be successful *at all* due to the effort needed to access information? We manipulate the degree to which exerting effort is likely to result in information by requiring that participants solve easy or difficult word search puzzles to see the answer to a trivia question. If the participant fails to solve a puzzle within a fixed time limit, they do not get to see the answer to the corresponding trivia question. Note that this manipulation also encompasses effort, like Experiments 1–2: difficult word search puzzles require more effort to solve *and* it is less likely that information will successfully be obtained.

Because we found few differences between Experiments 1–2 despite using different participant samples (student participant pool vs. Prolific), we returned to using a student participant pool in Experiment 3 for its greater convenience. We also used a new stimulus set of trivia questions to extend the overall generalizability of our results. Experiment 3 was preregistered at https://aspredicted.org/z873-7hs8.pdf.

### Methods

#### Participants.

We recruited 163 participants from a student participant pool at the University of New Hampshire. All participants completed the study online. An additional 35 participants were excluded for failing to pass three attention checks (described below). One additional participant was excluded for providing the same curiosity and confidence rating on all trials. One participant was also excluded due to data saving issues.

Our sample ranged from 18–25 years of age (*M* = 19). 82% of participants identified as women, 17% as men, and 1% as non-binary or genderqueer. Participants’ race/ethnicity were as follows: 84% White, 9% Multiracial and/or Multiethnic, 2% Asian, 2% Hispanic or Latino, 1% Black or African American, 1% Middle Eastern or North African, 1% Native Hawaiian or Pacific Islander.

Like Experiment 2, participants were randomly assigned to one of two task conditions: the forced-effort condition (*n* = 82) or the choice condition (*n* = 81). Our target sample size was 150, based on the same power analysis as Experiment 2.

#### Materials.

In Experiment 3, we used a new set of 20 trivia questions drawn from Galli et al. (2018), to test whether results from Experiments 1–2 generalized to a new stimulus set. We also developed a set of 20 word search puzzles—10 easy and 10 hard. In each word search puzzle, one four-letter word was hidden (unrelated to the trivia question or its answer). In easy word search puzzles, the word was hidden in a 5 × 5 grid. In hard word search puzzles, the word was hidden in a 9 × 9 grid.

We selected these word search puzzles based on a pretest. We pretested a larger set of 60 word search puzzles (5 × 5, 7 × 7, and 9 × 9) with an additional sample of 100 participants. In the pretest, participants were given an unlimited amount of time to find the word, then a fixed amount of time to click each letter to identify the word on the grid. We recorded—for correct answers only—the total time the participant took to both find the word and click each letter. Then, for the easy puzzles used in this experiment, we selected the ten 5 × 5 puzzles that the largest proportion of participants solved within 9 seconds total (all greater than 70% success rate). For the hard puzzles used in this experiment, we selected the ten 9 × 9 puzzles that the smallest proportion of participants solved within 9 seconds total (all less than 30% success rate). Nine seconds was selected as the cutoff somewhat arbitrarily, but not without justification: it provided a reasonable spread of difficulty levels (ranging from 10% to 96% success rate). Other nearby cutoffs would have yielded similar sets of “easy” and “hard” puzzles.

#### Procedure.

The procedure was similar to Experiments 1 and 2. Each participant completed 20 trivia trials. In the low-effort condition (10 questions) participants were informed that they would see a word search puzzle that over 70% of previous participants solved within 9 seconds. In the high-effort condition (10 questions) participants were informed that they would see a word search puzzle that less than 30% of previous participants solved within 9 seconds.

Critically, participants would only receive the answer to a question if they successfully solved the puzzle within the allotted time. Thus, there was a lower probability that participants would receive the answer to the trivia question on high-effort trials compared to low-effort trials. Participants were informed that each word search puzzle was randomly paired with a trivia question, so that whether the word search puzzle was easy or hard was completely unrelated to the trivia question itself.

After reading the instructions, participants were given a four-question multiple-choice comprehension quiz. Participants were required to answer all questions correctly to proceed (incorrect answers were indicated, and participants were given unlimited chances to try again). 82% of participants answered all four questions correctly on their first attempt. All participants passed the instructions quiz and proceeded to the main task within four attempts.

**Figure 3.**
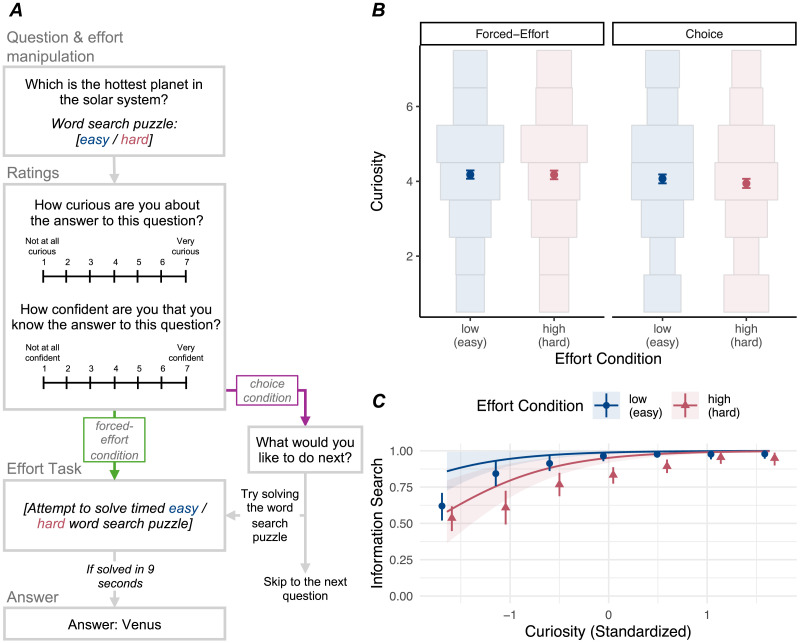
Experimental paradigm (A) and results (B, C) for Experiment 3. (B) Points indicate mean curiosity in each effort condition and task condition; error bars indicate 95% CIs. Histograms show the distribution of curiosity ratings within each effort condition and task condition. There was no evidence that effort condition affected curiosity. (C) Points indicate proportion of trials where a participant (in the choice condition) chose to engage in information search at each level of curiosity, error bars indicate 95% CIs. Lines (with 95% CIs) display model predictions from the reported mixed-effects model predicting information search as a function of curiosity and effort condition. Information search was more likely at higher levels of curiosity and lower levels of expected effort.

On each trial (see Figure 3), participants saw a single trivia question, with the associated effort condition (whether they would need to solve an easy or hard word search puzzle to see the answer). Then, participants rated curiosity and confidence. Next, participants in the forced-effort task condition attempted to solve the word search puzzle: they tried to find the word in the grid and click on each letter of the word within 9 seconds (with a 9-second countdown shown onscreen). Participants in the choice condition were given the option to choose what they wanted to do next: attempt the word search puzzle or skip to the next question (without seeing the answer). Finally, if participants solved the word search puzzle correctly within the 9-second time limit, they were immediately shown the answer to the trivia question.

After completing these tasks for all 20 trivia questions, participants completed the same attention checks and demographic questions as Experiments 1 and 2.

#### Analytic Approach.

Analyses were identical to Experiment 2. All analyses were all preregistered unless otherwise noted. Unlike Experiments 1–2, some participants failed to provide responses to some items. Specifically, there were 12 missing responses for curiosity (less than 1% of all responses), 10 missing responses for confidence (less than 1% of all responses), and 32 missing responses for information search in the choice condition (2% of all responses). For any analysis using a variable with missing responses, we excluded the missing trials. Note that if an information search decision was left blank, the participant automatically skipped to the next trial without completing the word search or seeing the trivia answer.

### Results

#### Manipulation Check.

Like Experiments 1 and 2, we tested whether participants were able to receive information more quickly in the low-effort condition compared to the high-effort condition. Looking only at trials where participants successfully solved the word search puzzle, participants were indeed faster on trials in the low-effort condition (*M* = 3.99 seconds, *SD* = 1.27) than they were on trials in the high-effort condition (*M* = 6.23 seconds, *SD* = 1.62). This difference was significant, *b* = −2.29, 95% CI [−2.43, −2.16], *χ*^2^(1) = 300.72, *p* < .001. This analysis was exploratory, but suggests that our manipulation of effort did influence the time people spent on the word search task.

We also verified that participants were more likely to successfully solve low-effort word search puzzles compared to high-effort puzzles. In an exploratory analysis, we fit a mixed-effects model predicting successful puzzle solutions as a function of effort condition. Participants were much more likely to solve puzzles in the low-effort condition compared to the high-effort condition, OR = 53.28, 95% CI [33.83, 83.91], *χ*^2^(1) = 286.53, *p* < .001. Participants solved 95% of attempted puzzles in the low-effort condition and only 42% of attempted puzzles in the high-effort condition.

#### Effort and Curiosity.

As in Experiment 2, we first tested the effect of effort on curiosity in the forced-effort condition. We found no evidence that curiosity was affected by expected effort, *β* = −0.01, 95% CI [−0.09, 0.08], *χ*^2^(1) = 0.02, *p* = .88 (see Figure 3). The average participant-level curiosity rating in the low-effort condition was 4.18 (*SD* = 0.93), while the average curiosity rating in the high-effort condition was 4.17 (*SD* = 0.99). To further test the effect of effort on curiosity, we conducted a Bayesian version of the above analysis. The Bayes Factor suggested that the data were much more likely under the null model than the alternative model (BF = 120.26). Moreover, the posterior estimate for the fixed effect of effort in the alternative model was largely concentrated around zero (median = −0.01, 95% HDI [−0.09, 0.08]). This suggests that participants’ curiosity is unlikely to be affected by expected effort, even when there was a possibility of failing to gain information.

Reported in the Supplementary Materials, we conducted preregistered analyses asking whether the effect of effort was moderated by participant-reported confidence. In Experiment 2, but not Experiment 1, we found an effect of effort at the highest levels of confidence. We did not replicate this effect in Experiment 3, suggesting that this indeed may only arise when rating curiosity in extreme high-confidence situations (e.g., after already seeing the first letter of the answer, as was the case in Experiment 2).

Next, we investigated whether the effect of effort on curiosity varied across task conditions (forced-effort vs. choice). We added task condition to the model predicting curiosity as a function of effort. There was no evidence that interaction between effort and task condition made a significant contribution to the model, *β* = 0.06, 95% CI [−0.07, 0.18], *χ*^2^(1) = 0.83, *p* = .36. In the choice condition, the participant-level average curiosity rating was 4.08 (*SD* = 0.96) for low-effort trials and 3.95 (*SD* = 0.88) for high-effort trials (see Figure 3). In sum, there was no evidence—across conditions—that effort affected curiosity. In addition, unlike Experiment 2, we found no evidence that curiosity was affected by task condition, *β* = −0.09, 95% CI [−0.24, 0.05], *χ*^2^(1) = 1.59, *p* = .21.

#### Effort and Information Search.

Replicating Experiment 2, expected effort robustly affected information search: participants were more likely to seek information when expected effort was low compared to high (see Figure 3). On average, participants sought information on 9.08 out of 10 low-effort trials (*SD* = 1.73) and 8.03 out of 10 high-effort trials (*SD* = 2.31). We fit a mixed-effects logistic regression model predicting information search, with effort as a fixed effect. There was evidence for a significant effect of effort, OR = 3.94, 95% CI [1.84, 8.44], *χ*^2^(1) = 13.10, *p* < .001, with higher odds of information search in the low-effort condition compared to the high-effort condition. When curiosity was added to this model (exploratory; as a fixed effect and by-participant random slope), there was also an effect of curiosity, OR = 5.03, 95% CI [2.99, 8.47], *χ*^2^(1) = 32.51, *p* < .001, with higher curiosity predicting greater odds of information search. Thus, subjective curiosity and effort both independently predicted decisions to seek information.

Notably, in this experiment (unlike Experiment 2), participants chose to seek information on a high proportion of trials. Even when curiosity was rated a “1” (the minimum), participants chose to seek information on 57% of trials (compared to 96% of trials for a “7” curiosity rating). In addition, 31% of participants chose to seek information on every trial. This likely reflects that word search puzzles are fun, providing an additional motivation for participants to complete the effort task. Thus, it is notable that both self-reported curiosity and manipulated effort still impact participants’ information search decisions, even for tasks that might generally attract engagement.

As preregistered, we also investigated whether confidence mediated or moderated the effect of effort on information search and self-reported curiosity. These analyses are reported in the Supplementary Materials. Though people rated their confidence as significantly higher when effort was lower (i.e., for easy puzzles over hard puzzles), this did not mediate the effect of effort on information search. Moreover, there was no evidence that effort interacted with confidence in predicting information search or curiosity.

### Discussion

Experiment 3 tested whether expected effort affects curiosity when there is a possibility that information search will fail. We again found no evidence that expected effort affected self-reported curiosity—people were no more or less curious when they would need to exert a high degree of effort to obtain information compared to a lower degree of effort. Moreover, similar to Experiment 2, we found that expected effort did affect information search, providing evidence that our manipulation was effective.

Notably, there are several reasons that participants could fail to receive information in this experiment—because of the effort required, but also because of limitations in skill level, variation in puzzle difficulty, and so on. Moreover, by using a socially-relevant manipulation of effort (past participants’ solution rates), we may have introduced an additional motivation for participants to seek information—to demonstrate their own competence relative to other, previous participants (see Festinger, 1954). Thus, while we do not precisely isolate effort in this experiment, the results are aligned with Experiments 1–2. In addition, we show in the Supplementary Materials that participant skill level does not moderate the effect of effort condition on curiosity. Thus, taken together, these studies collectively suggest that expected effort affects information search, but not necessarily curiosity.

## GENERAL DISCUSSION

In this work, we tested whether curiosity is influenced by the amount of effort required to gain new information. Across three preregistered and well-powered experiments, we found very little evidence that the subjective experience of curiosity (measured through self-report) was influenced by effort. In fact, Bayesian analyses consistently suggested that the data were more likely under the null hypothesis: that effort *does not* affect curiosity. This was true both when information was guaranteed to be received with sufficient effort (Experiments 1–2) and when information search could fail to result in information (Experiment 3).

There was one exception to the above results. In Experiment 2, when people were highly confident that they already knew the answer, they reported higher curiosity when less effort was required to obtain the answer (15 vs. 60 key presses). This could reflect people’s preference to seek information that will confirm their beliefs (see Klayman & Ha, 1987). It is especially notable that this effect only arose in Experiment 2, where participants rated curiosity and confidence after revealing the first letter of the answer. High confidence ratings may have reflected that the participant’s initial guess about the answer was supported by the first revealed letter, and thus participants may have been especially motivated to confirm their guess. In Experiments 1 and 3, where participants rated confidence and curiosity without any hints about the answer, we did not find the same effect. Therefore, we expect that this result would only generalize to contexts where participants are forced to judge their curiosity about an answer that they have good reason to believe they already know—and even then, the effect of effort was small. Thus, the effect of expected effort on curiosity is highly limited and we believe unlikely to arise in real-world learning environments.

Despite finding no evidence for an effect of expected effort on subjective curiosity, we found in Experiments 2–3 that expected effort affected participants’ decisions to seek information. Specifically, participants were more likely to opt out of a high-effort task (thus foregoing the opportunity to gain information) compared to a low-effort task. This validates our manipulation by showing that effort has an influence on behavior, even if not on self-reported curiosity. However, it also raises questions about the precise relations between effort, curiosity, and information search. Specifically, prior work has sometimes measured curiosity through information search decisions (Blanchard et al., 2015; Dubey & Griffiths, 2020; FitzGibbon et al., 2019; Hsee & Ruan, 2016; Jirout & Klahr, 2012; Kobayashi et al., 2019; Subbotsky, 2010; Wang & Hayden, 2019). Here, however, we find that information search is affected by effort while self-reported curiosity is not. What explains this disconnect?

Information search decisions are likely made by balancing the benefits of information search (e.g., satisfying curiosity, having fun) against the costs (e.g., exerting effort, taking longer to complete the experiment). Thus, information search decisions may partly reflect curiosity—but they may also reflect other causes. In our case, the costs of information search are affected by our manipulation of effort: a low-effort task makes information search less costly than a high-effort task. Because of this, information search cannot be interpreted as a clear indicator of curiosity—if people choose actions by weighing costs (e.g., effort) and benefits (e.g., satisfying curiosity), information search will be affected by the effort manipulation (by way of the increased costs) even if the effort manipulation does not affect curiosity.

At the same time, curiosity self-reports may be limited if people do not have accurate introspective access to their own curiosity. Therefore, both information search and self-report judgments are imperfect measures of the construct we wish to study: “true” curiosity. As a result, we conclude that effort does not impact subjective self-assessments of curiosity, even if it may affect “true” curiosity. It also remains possible that information search and self-reported curiosity could measure different types or forms of curiosity—one of which is affected by effort, and the other of which is not. Regardless, it is notable that information search can come apart from subjective judgments of curiosity. The factors that influence information search might not always affect subjective feelings of curiosity, and vice versa. This implies that future work that measures *both* curiosity and information search will likely provide deeper insight into the mechanisms underlying curiosity.

Notably, the population studied here was adults in the United States (primarily college students who were young adults in Experiments 1 and 3). The results found with college students in Experiments 1 and 3 were similar to the results found with a broader population of adults in Experiment 2. However, further work is needed to understand how the effect of effort on curiosity might vary across cultures, contexts, or age. For example, prior research suggests that curiosity changes across development, such that children’s curiosity and older adults’ curiosity may be elicited through different mechanisms than young adults’ curiosity (Fastrich et al., 2024; Liquin et al., 2021, 2025). Thus, it might be fruitful for future work to study how the effect of effort on curiosity varies across the lifespan. In the following sections, we review two other promising areas for future work.

### Effort and the Learning Process

First, these findings shed light on broader questions about what triggers curiosity. Recent work has proposed that curiosity is tightly linked to the process of learning. Specifically, people are more curious when they expect to learn (Liquin et al., 2020; Liquin & Lombrozo, 2020), when they feel they are on the verge of learning (Metcalfe et al., 2020), and when their learning is progressing rapidly (Poli et al., 2022, 2024). If curiosity is sensitive to the process of learning, we might expect that practical hurdles to learning—for example, that a high amount of effort is required to obtain novel information—would also matter for determining that individual’s level of curiosity. Especially in Experiment 3, when information is only delivered upon *successful* completion of a word search puzzle, one should judge that learning is less likely to occur on high-effort trials (hard word search puzzles) than low-effort trials (easy word search puzzles). However, we find no evidence that effort affects self-reported curiosity, even in Experiment 3.

One possible interpretation of these results is that curiosity is primarily sensitive to the “idealized” process of learning—a representation of the learning that is expected to occur *if and when* information is received (e.g., assuming that the effort required to obtain new information is immaterially low, or that one has high capacity to overcome the relevant hurdles)—rather than the “actual” process of learning—a representation of the learning that is actually about to occur. If expectations about *actual* learning shape curiosity, the effort manipulation should impact curiosity ratings because a higher degree of expected effort makes learning less attainable. Given that we find no evidence for an effect of effort on curiosity, we tentatively suggest that curiosity might primarily be sensitive to an idealized representation of the learning process, where successful learning is taken as a given.

Further work is needed to provide a strong test of this proposal. In particular, by focusing on trivia questions, the present research provides only a narrow window into the learning process. Participants in our studies learned individual, trivial facts. In contrast, prior work on learning progress has investigated curiosity and information search in dynamic, trial-and-error learning tasks, rather than in response to static trivia stimuli (Poli et al., 2022; Ten et al., 2021; see also Hsiung et al., 2023). Moreover, we did not explicitly assess participants’ prior knowledge or later memory, meaning we do not know what participants actually learned. Thus, more work is needed to understand how expected effort interacts with people’s learning over time to produce feelings of curiosity. It remains possible that our paradigm does not capture the relevant aspects of the learning process that might be affected by expected effort.

Other manipulations of effort might also shed further light on how effort affects curiosity. Here, we manipulated effort using a task that was completely divorced from the learning process. In contrast, real-world effort is often integrated with the process of learning. For example, reading a book to find the answer to a question requires effort, but also provides engaging, topic-relevant information. While our design allows us to cleanly test whether effort affects curiosity (and mimics some real-world information search tasks, like driving 30 minutes to the library to pick up an informative book), divorcing information search from the learning process may have led participants to be disengaged from the task—perhaps obscuring any effect of effort on curiosity. Moreover, in many real-world learning tasks, effort is not arbitrary. For example, an organic chemistry class is effortful not because it requires effortful engagement on arbitrary assignments—but because effort is required to learn the challenging material. Thus, a high degree of effort can signal that learning itself is particularly difficult, and thus perhaps, that learning progress is slow. In cases where effort is a signal of likely learning progress, there is reason to think that effort *would* impact curiosity.

### Avoiding (or Seeking) Effort

The present findings also shed light on how curiosity intersects with people’s desire to avoid effort. Prior work has shown that people are more willing to complete effortful or time-consuming tasks to receive a trivia answer when they are more curious about the corresponding trivia question (Dubey & Griffiths, 2020; Kang et al., 2009; Spitzer et al., 2025). We replicated these effects in Experiments 2 and 3: participants’ reported level of curiosity predicted their decisions to engage in time-consuming and/or effortful tasks to gain information. However, subjective curiosity ratings were not affected by *how* time-consuming or effortful information search would be.

This contradicts the predictions of “delay discounting” and “effort discounting” (Ainslie, 1975; Frederick et al., 2002; Hull, 1943; Prévost et al., 2010; Rachlin et al., 1991; Westbrook et al., 2013). If people value information less when more effort or time is required to obtain it, we would expect lower levels of curiosity at higher levels of expected effort. Indeed, Molnar and Golman (2024) found that people were less likely to engage in effortful information search when information would be received after a delay rather than immediately—consistent with delay discounting. Here, we find similar results for information search decisions, but not for self-report judgments of curiosity. Moreover, another study that measured curiosity through self-report (Noordewier & van Dijk, 2017) found no evidence that a time delay affected curiosity. Taken together, these findings suggest that delay and effort affect explicit decisions to seek information, but not the subjective experience of curiosity itself. If delay and effort do discount the value of information, more research is needed to understand the mechanisms by which discounting affects information search but not subjective feelings of curiosity.

It is also important to reiterate that participants could have chosen to engage in (or avoid) the effort task in Experiments 2 and 3 for reasons other than expected effort or curiosity—especially in Experiment 3, participants may have simply wanted to attempt puzzles (e.g., reflecting need for cognition, see Cacioppo & Petty, 1982). Similarly, participants may have avoided the effort task in order to end the experiment earlier. Reassuringly, very few participants opted out of the effort task on all trials, and people were more likely to engage in the effort task when they were more curious. This suggests that curiosity plays *some* role in motivating information search decisions.

Relatedly, some have suggested that people sometimes *seek* rather than avoid effort (Inzlicht et al., 2018; Job et al., 2024). In our studies, people seemed to be generally motivated to avoid effort—participants were more likely to opt out of the effort task when it was high-effort compared to low-effort. However, it is unclear how effort would shape curiosity in situations where effort is viewed as desirable or valuable. Effort can be sought out for several reasons (see Inzlicht et al., 2018; Job et al., 2024): for example, when alternatives are too boring (e.g., doing nothing; Embrey et al., 2024; Wu et al., 2023). Moreover, effort can signal an opportunity for learning progress or increased mastery: tasks that require moderate effort can be learned, while those that require little effort have already been mastered. Supporting this possibility, people are more engaged with cognitively effortful tasks when they provide an opportunity for learning progress (Sayalı et al., 2023). As mentioned above, in cases where effort is a signal of learning progress, we might expect effort to influence curiosity. Thus, future research might attempt to extend our investigation using an information search task where effort is desirable rather than aversive, shedding further light on when and why effort might shape curiosity.

Considering recent developments in AI, it is more important now than ever before to understand the effect of effort on curiosity. Prior work has shown that easy access to information via Google might inflate people’s confidence in their own knowledge (Fisher et al., 2015), and emerging evidence shows that this effect may extend to AI use, as well (Fisher & Oppenheimer, 2021; Hamilton et al., 2025). While there are several explanations for these findings, some suggest that the sense of fluency that accompanies effort-free access to information plays a key role (e.g., Hamilton & Qi, 2023). If low-effort information search increases one’s confidence in their own knowledge, we might also expect downstream effects on curiosity. Here, in contrast, we found that the degree of effort required to obtain information had little effect on confidence (with the exception of Experiment 3, see Supplementary Materials) or on self-reported curiosity. There are many methodological differences between our tasks and those used to study AI/internet use, making it difficult to pinpoint the source of these differing results. However, in future work, it would be valuable to test whether variation in the effort associated with AI/internet use shapes curiosity, in addition to confidence.

### Conclusion

In sum, we find that people are less likely to pursue information search when higher levels of effort are required, but this does not dampen their feelings of curiosity. Many learning problems, like understanding the nature of the human mind, require substantial time and effort to make even incremental progress. It takes years to pursue a Ph.D. or conduct and publish new research, yet we still feel curious about the mysteries of the human mind. Here, we show that this could be a general feature of curiosity: even for simple trivia-question tasks, subjective feelings of curiosity are unlikely to be affected by the effort needed to obtain new information.

## ACKNOWLEDGMENTS

Thank you to Sydney Staples, Angel Eaton, and Zach Gabriel for their assistance with methodology and data collection, and to Casey Roark and members of the Exploration, Learning, and Mind lab at the University of New Hampshire for comments on earlier versions of this work.

## FUNDING INFORMATION

This research was supported by internal funds from the University of New Hampshire.

## AUTHOR CONTRIBUTIONS

E.G.L.: Conceptualization; Data curation; Formal analysis; Investigation; Methodology; Project administration; Validation; Visualization; Writing – original draft; Writing – review & editing.

## DATA AVAILABILITY STATEMENT

All data and code are available at https://osf.io/js7x2. Experiment 1 was preregistered at https://aspredicted.org/z525-3njf.pdf. Experiment 2 was preregistered at https://aspredicted.org/bb6v-xdh7.pdf. Experiment 3 was preregistered at https://aspredicted.org/z873-7hs8.pdf.

## Note

^1^ We used the default weakly informative priors as set by the stan_glmer function in the rstanarm R package (Goodrich et al., 2020).

## Supplementary Material


