# Supplementary material for: More Time and Effort, Same Curiosity: Expected Effort Does Not Impact Curiosity
Source: Open Mind (Camb). 2026 Mar 15;10:391–414. doi: 10.1162/OPMI.a.342 (PMC13056335; doi:10.1162/OPMI.a.342)
Supplement: Supplementary file 1 [file opmi-10-391-s001.pdf]

Supplementary Materials for

"More time and effort, same curiosity: Expected effort does not impact curiosity"

Emily G. Liquin

Department of Psychology, University of New Hampshire

emily.liquin@unh.edu

Supplementary analyses, Experiment 1

Does effort affect confidence and answer satisfaction?

In exploratory analyses, we tested whether confidence (rated upon seeing the question) or satisfaction (rated upon seeing the answer) were affected by effort. For each, we fit a mixed-effects model, with effort condition as a fixed effect, by-participant random intercepts, and by-participant random slopes for effort condition. There was no evidence for a significant effect of effort condition on either confidence, $\beta = 0.003$, 95% CI [-0.05, 0.05], $\chi^2(1) = 0.02$, $p = .90$, or satisfaction, $\beta = 0.02$, 95% CI [-0.04, 0.08], $\chi^2(1) = 0.41$, $p = .52$.

Does the effect of effort vary across levels of confidence?

Next, we conducted exploratory analyses investigating whether the effect of effort varied across levels of confidence. When participants rated their confidence as high, they likely already knew the answer to the question—meaning they were unlikely to be curious. Indeed, prior research finds an inverted-U-shaped relation between confidence and curiosity, with the highest levels of curiosity at moderate levels of confidence (Kang et al., 2009; Ten et al., 2025). We replicate this effect here: We fit an exploratory mixed-effects regression model predicting curiosity, with fixed effects for the linear and quadratic effects of confidence, by-participant and by-trivia question random intercepts, and by-participant slopes for linear and quadratic

confidence. There was evidence for both a linear effect of confidence, $\beta = 0.56$, 95% CI [0.46, 0.65], $\chi^2(1) = 81.14$, $p < .001$, and a quadratic effect of confidence, $\beta = -0.33$, 95% CI [-0.38, -0.27], $\chi^2(1) = 85.84$, $p < .001$. Curiosity was highest at moderate-to-high levels of confidence.

We wondered whether an effect of effort on curiosity might only arise at certain levels of confidence—for example, when confidence is low or moderate. When confidence is high, effort might not impact curiosity simply because the participant has no reason to be curious. Thus, it is important to test whether an effect of effort on curiosity only arises at specific levels of confidence.

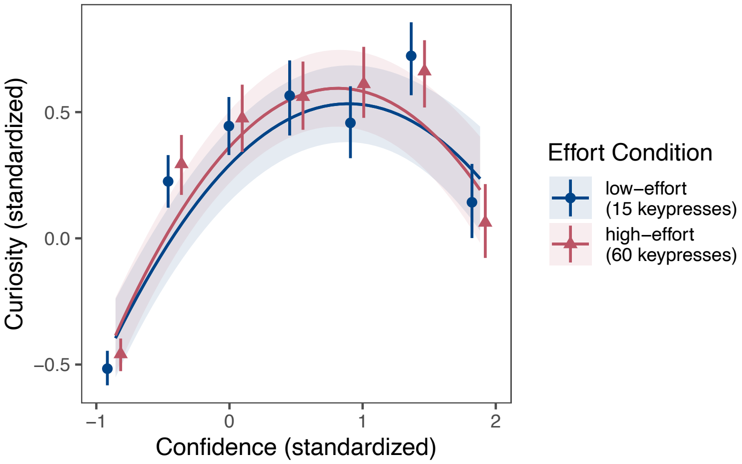

Figure S1. Experiment 1 curiosity ratings, plotted as a function of confidence ratings and effort condition. Points indicate mean curiosity at each level of confidence; error bars indicate 95% CIs. Lines (with 95% CIs) display model predictions from the reported mixed-effects model predicting curiosity as a function of confidence and effort condition. Notably, the effect of effort condition on curiosity was not moderated by confidence.

However, we found no evidence that effort interacted with the linear or quadratic effect of confidence (see Fig. S1). As an exploratory analysis, we added effort condition to the model above as a fixed effect (in interaction with linear/quadratic confidence) and a by-participant

random slope.[1] There was no evidence for an interaction between effort and the linear effect of confidence, β = -0.03, 95% CI [-0.10, 0.04], $\chi^2(1) = 0.63$, $p = .43$, or between effort and the quadratic effect of confidence, β = 0.05, 95% CI [-0.02, 0.12], $\chi^2(1) = 1.91$, $p = .17$. Thus, there was no evidence that the effect of effort on curiosity varied across levels of confidence. This suggests that the null result is generalizable to a range of questions, independent of participants' prior confidence that they know the answer.

Effort across trials

In an additional exploratory analysis, we tested whether the effect of effort on curiosity varied across trials throughout the task. One might expect that the effect of effort only emerges after substantial experience encountering high- and low-effort tasks. We fit a mixed-effects model predicting curiosity, with fixed effects for effort condition, trial number, and their interaction. The model also included by-participant and by-question random intercepts, as well as by-participant random slopes for effort condition and trial number. There was no evidence for an interaction between effort condition and trial number, β = 0.002, 95% CI [-0.002, 0.01], $\chi^2(1) = 1.02$, $p = .31$. Thus, the effect of effort did not significantly vary over the course of the task.

Supplementary analyses, Experiment 2

Does effort affect confidence?

Replicating the results of Experiment 1, there was no evidence for a significant effect of effort condition on confidence in Experiment 2, β = 0.01, 95% CI [-0.04, 0.07], $\chi^2(1) = 0.32$, $p = .57$.

Does the effect of effort vary across levels of confidence?

[1] We did not include random slopes for the interaction between effort condition and linear/quadratic confidence, as this model resulted in a singular fit. Thus, the fitted model assumes a population-level interaction effect that does not vary in magnitude across participants.

Replicating Experiment 1 and prior work, we found that curiosity was related to confidence in the forced-effort condition, with high curiosity at moderate levels of confidence. In an exploratory analysis, there was evidence for both a linear effect of confidence on curiosity, $\beta = 0.10$, 95% CI [0.01, 0.19], $\chi^2(1) = 4.36$, $p = .04$, and a quadratic effect of confidence on curiosity, $\beta = -0.36$, 95% CI [-0.45, -0.27], $\chi^2(1) = 45.50$, $p < .001$. Thus, curiosity was highest at moderate-to-high levels of confidence.

As in Experiment 1, we conducted an exploratory analysis investigating whether effort condition interacted with confidence (in the forced-effort condition).[2] We found evidence for an interaction between effort condition and the quadratic effect of confidence, $\beta = 0.12$, 95% CI [0.02, 0.22], $\chi^2(1) = 5.94$, $p = .01$. Following up on this interaction, we calculated the estimated marginal mean contrast (using the emmeans R package; Lenth, 2023) between the low-effort condition and the high-effort condition at three levels of confidence: the minimum, midpoint, and maximum of the seven-point rating scale. The effort contrast was only significant at the highest levels of confidence (see Fig. S2): when participants indicated that they were very confident that they knew the answer (7 out of 7), their curiosity was 0.15 standardized units higher in the low-effort condition than the high-effort condition, $t(1548) = 2.60$, $p = .01$. Thus, people were more curious when expecting lower levels of effort compared to higher levels of effort, but only when they were already highly confident they knew the answer.

---

[2] Unlike Experiment 1, we did not include by-participant random slopes for effort condition in this analysis due to model convergence issues. This can occur in particularly complex models when observations are not sufficient to estimate the random effects. We initially followed the recommendation of Barr et al. (2013) to remove the correlation between random effects, but this led to a solution with singular fit (with no by-participant variation in the effect of effort condition). Therefore, in our main analysis, we removed the by-participant random slope for effort condition but retained the correlation between random effects. The key difference is that the initially planned model assumes that effort condition might have different effects for different participants, but the final analysis assumes no such variability. Notably, these slight variations in random effects structure had no major impact on the reported fixed effects.

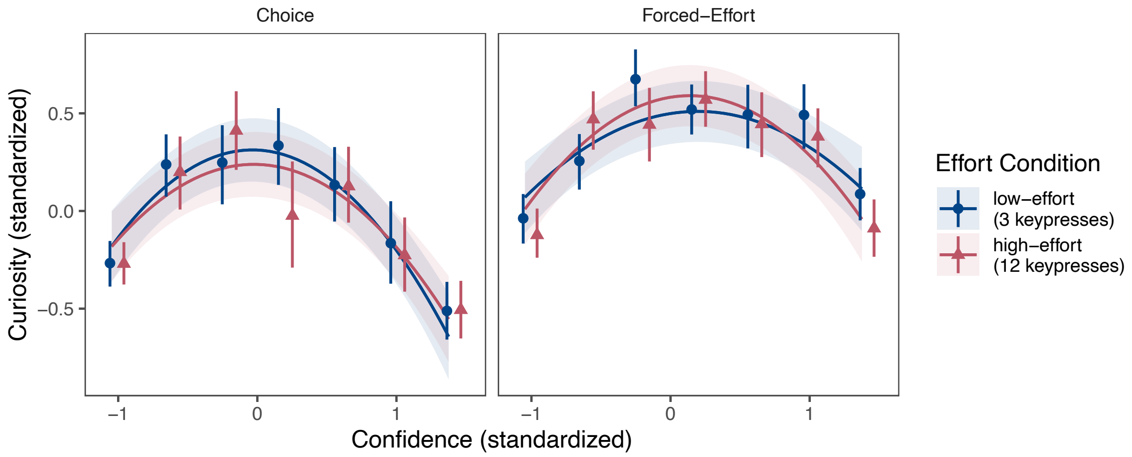

Figure S2. Experiment 2 curiosity ratings, plotted as a function of confidence ratings, effort condition, and task condition. Points indicate mean curiosity at each level of confidence; error bars indicate 95% CIs. Lines (with 95% CIs) display model predictions from the reported mixed-effects model predicting curiosity as a function of confidence, effort condition, and task condition. Notably, effort condition only affected curiosity at maximal levels of confidence in the forced-effort condition.

We also tested whether the interaction between effort condition and confidence varied across task conditions (forced-effort vs. choice), in an exploratory analysis. As reported above, there was a two-way interaction between effort and the quadratic effect of confidence in the forced-effort condition. However, there was no evidence for an interaction between effort and the quadratic effect of confidence in the choice condition, β = -0.08, 95% CI [-0.21, 0.04], $\chi^2(1)$ = 1.81, p = .18. The three-way interaction was significant, β = -0.21, 95% CI [-0.36, -0.05], $\chi^2(1)$ = 6.70, p = .01. Thus, higher levels of effort led to decreased curiosity at high levels of confidence, but only when participants had no choice but to pursue the answer (see Fig. S2).

Effort across trials

Replicating Experiment 1, there was no evidence that the effect of effort varied across trials, β = 0.002, 95% CI [-0.01, 0.01], $\chi^2(1)$ = 0.30, p = .58.

Supplementary analysis, Experiment 3

Confidence, effort, and curiosity

As in the previous studies, we found that curiosity was related to confidence, with high curiosity at moderate levels of confidence. In the forced-effort condition, there was evidence for both a linear effect of confidence, $\beta = 0.11$, 95% CI [0.04, 0.19], $\chi^2(1) = 8.10$, $p = .004$, and a quadratic effect of confidence, $\beta = -0.16$, 95% CI [-0.20, -0.11], $\chi^2(1) = 30.83$, $p < .001$.

However, effort did not interact with confidence. We fit a mixed-effects model predicting curiosity, with fixed effects for effort condition interacting with confidence (linear and quadratic). This model included by-participant and by-question random intercepts, as well as random slopes for the linear effect of confidence and effort. An equivalent model with random slopes for the quadratic effect of confidence led to singular fit. There was no evidence for an interaction between effort condition and the linear effect of confidence, $\beta = -0.03$, 95% CI [-0.14, 0.08], $\chi^2(1) = 0.29$, $p = .59$, or the quadratic effect of confidence, $\beta = 0.05$, 95% CI [-0.03, 0.13], $\chi^2(1) = 1.28$, $p = 26$. This suggests that curiosity was unaffected by effort across levels of confidence (see Fig. S3).

There was also no evidence for three-way interactions between effort, task condition, and the linear effect of confidence, $\beta = -0.01$, 95% CI [-0.17, 0.15], $\chi^2(1) = 0.02$, p = .89, or the quadratic effect of confidence, $\beta = 0.02$, 95% CI [-0.10, 0.15], $\chi^2(1) = 0.14$, p = .71. In sum, there was no evidence—in any condition, at any level of confidence—that effort condition affected curiosity.

Confidence, effort, and information search

As preregistered, we tested whether the effect of effort condition on information search was mediated or moderated by confidence. Unlike Experiments 1 and 2, we found that confidence was slightly higher for low-effort questions compared to high-effort questions, $\beta =$

0.14, 95% CI [0.04, 0.24], $\chi^2(1) = 7.94$, $p = .005$. This suggests that confidence could be a potential mediator of the effect of effort condition on information search. However, we then fit a logistic mixed-effects model predicting information search, with fixed effects for confidence and effort condition, by-participant random intercepts and slopes, and by-question random intercepts. The effect of effort condition on information search remained significant even when controlling for confidence, $OR = 4.02$, 95% CI [1.82, 8.85], $\chi^2(1) = 12.67$, $p < .001$. Thus, confidence does not fully mediate the effect of effort on information search.

It is puzzling that confidence was higher for low-effort than high-effort questions in Experiment 3, but not in Experiments 1 or 2. Notably, in Experiment 3, the labels used to convey effort were "easy" and "hard"—corresponding to the difficulty of the to-be-solved word search puzzle. In Experiments 1 and 2, the effort labels corresponded to the number of keypresses required (e.g., 15 or 60). We suspect that a small number of participants may have misinterpreted the "easy" and "hard" labels as referring to the trivia question rather than the word search—leading to higher confidence ratings in response to "easy" labels (low-effort) than "hard" labels (high-effort). Indeed, inspecting the by-participant random slopes for effort condition, there are a few participants with noticeably strong positive slopes, while most are centered around zero. It would therefore be useful for future research to replicate these results with different condition labels.

We also found that the effect of effort condition on information search did not vary across levels of confidence. We fit a logistic mixed-effects model predicting information search, with confidence as a fixed effect, by-participant random intercepts and slopes for confidence, and by-question random intercepts. We deviated from our preregistration in not including a quadratic effect for confidence—while confidence is quadratically related to curiosity, there was no

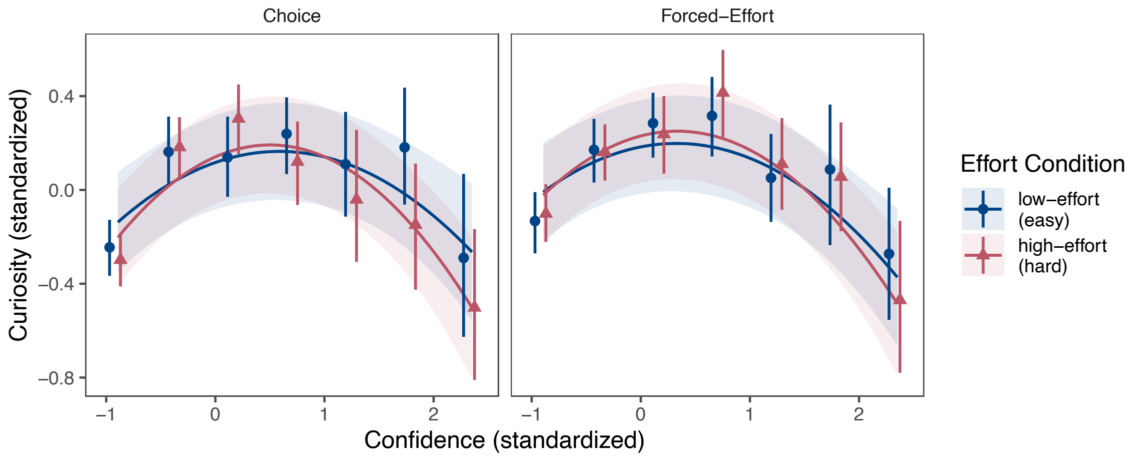

Figure S3. Experiment 3 curiosity ratings, plotted as a function of confidence ratings, effort condition, and task condition. Points indicate mean curiosity at each level of confidence; error bars indicate 95% CIs. Lines (with 95% CIs) display model predictions from the reported mixed-effects model predicting curiosity as a function of confidence, effort condition, and task condition. Notably, the effect of effort condition on curiosity was not moderated by confidence.

evidence that the quadratic effect of confidence improved the model's ability to predict information search, *OR* = 0.85, 95% CI [0.67, 1.06], $\chi^2(1) = 1.96$, $p = .16$. Then, we refit this model adding effort as a fixed effect (in interaction with confidence) and by-participant random slope. There was no evidence for a significant interaction between confidence and effort condition, *OR* = 1.12, 95% CI [0.65, 1.95], $\chi^2(1) = 0.17$, $p = .68$. Thus, there was no evidence that the effect of effort on information search varied across levels of confidence.

Effort across trials

Like Experiments 1 and 2, we tested whether the effect of effort condition varied across trials. There was no evidence for an interaction between effort condition and trial number, β = -0.00001, 95% CI [-0.01, 0.01], $\chi^2(1) = 1.95$, $p = .16$. Thus, there was no evidence that the effect of effort varied across trials.

Does word-search skill moderate the effect of effort?

One possible concern with the design of Experiment 3 is that some participants may lack the skill to successfully solve word search puzzles. If so, our manipulation of effort may fail to induce varying expectations of effort. This is because particularly unskilled participants might decline to exert *any* effort—meaning they should expect both easy and hard word-search puzzles to require low levels of effort. If there is an effect of effort on curiosity, we might expect this to only emerge for highly skilled participants.

To explore this possibility, we calculated the proportion of attempted word search puzzles that each participant solved successfully. This provides us an estimate of participant skill (M = 0.69, SD = 0.13). Then, we asked whether skill level moderated the effect of effort condition on curiosity. We fit a mixed-effects regression model predicting curiosity, with fixed effects for effort condition, participant skill, and their interaction. The model also included by-participant and by-question random intercepts, as well as by-participant random slopes for effort condition. There was no evidence for an interaction between effort condition and participant skill, β = -0.01, 95% CI [-0.07, 0.05], $\chi^2(1) = 0.11$, p = .74, nor was the effect of participant skill significant in a model excluding the interaction term, β = 0.03, 95% CI [-0.04, 0.11], $\chi^2(1) = 0.78$, p = .38. Therefore, the effect of effort condition on curiosity—and curiosity itself—was consistent across participant skill levels.

References

Barr, D. J., Levy, R., Scheepers, C., & Tily, H. J. (2013). Random effects structure for confirmatory hypothesis testing: Keep it maximal. *Journal of Memory and Language*, *68*(3), 255–278. https://doi.org/10.1016/j.jml.2012.11.001

Kang, M. J., Hsu, M., Krajbich, I. M., Loewenstein, G., McClure, S. M., Wang, J. T., & Camerer, C. F. (2009). The wick in the candle of learning: Epistemic curiosity activates reward circuitry and enhances memory. *Psychological Science*, *20*(8), 963–973. https://doi.org/10.1111/j.1467-9280.2009.02402.x

Lenth, R. V. (2023). *emmeans: Estimated Marginal Means, aka Least-Squares Means*. https://CRAN.R-project.org/package=emmeans

Ten, A., Oudeyer, P.-Y., Sakaki, M., & Murayama, K. (2025). The curious U: Integrating theories linking knowledge and information-seeking behavior. *Open Mind*, *9*, 1763–1785.
